# Gadobutrol-enhanced magnetic resonance imaging of meningeal carcinomatosis: case report with emphasis on early diagnosis

**DOI:** 10.1186/s12883-016-0683-3

**Published:** 2016-09-01

**Authors:** Xinfa Pan, Yongfu Lu, Liang Wen, Xiujue Zheng, Yuehui Ma

**Affiliations:** 1 Department of Neurosurgery, The First Affiliated Hospital, College of Medicine, Zhejiang University, Hangzhou, 310003 Zhejiang China; 2 Department of Pathology, The First Affiliated Hospital, College of Medicine, Zhejiang University, Hangzhou, 310003 Zhejiang China

**Keywords:** Meningeal carcinomatosis, Diagnosis, Gadobutrol, Case report

## Abstract

**Background:**

Timely diagnosis of meningeal carcinomatosis is often difficult even with the assistant of magnetic resonance imaging examination, cerebrospinal fluid analysis, or both. To the best of our knowledge, gadobutrol-enhanced MRI has not been reported in the diagnosis of meningeal carcinomatosis. Here we present two cases where meningeal carcinomatosis was identified on gadobutrol-enhanced magnetic resonance imaging.

**Case presentation:**

We identified two cases of meningeal carcinomatosis who had been diagnosed with malignant tumors several years ago. Both patients presented with progressive headache and seizures. Gadopentetate dimeglumine-enhanced magnetic resonance imaging of the brain was performed and did not detect any abnormality of meninges. Lumbar puncture was performed repeatedly, but cerebrospinal fluid cytology showed no evidence of malignant cells. Finally the gadobutrol-enhanced magnetic resonance imaging detected the meningeal metastasis, and supported the diagnosis of meningeal carcinomatosis.

**Conclusion:**

Gadobutrol provides higher lesion conspicuity and enhances lesion detection in meningeal metastasis compared with gadopentetate dimeglumine. Our observation is a cue to analyze the accuracy in the diagnosis of meningeal carcinomatosis, and presents a choice that may facilitate early diagnosis.

## Background

Meningeal carcinomatosis (MC) which results from the multifocal seeding of meninges by malignant cells is diagnosed in 1–5 % of patients with malignant tumors [1]. MC is much more frequently observed in patients diagnosed with lung and breast cancer, melanoma as well as lymphoma and leukemia. The clinical presentation of MC, such as headache and nerve palsy is non-specific, but the condition deteriorates very quickly. The gold diagnosis standard is cerebrospinal fluid (CSF) cytology, and the neuroradiographic examination with gadolinium magnetic resonance imaging (gdMRI) facilitates the diagnosis [2]. However timely diagnosis of MC is often difficult even with the assistant of MRI examination, CSF analysis, or both. Radiotherapy and chemotherapy are the main therapeutic approaches, and the supportive treatment is also very important for symptom relief. The prognosis for MC is poor and the median survival is between 4 and 6 weeks [3]. Early diagnosis and intervention help improve quality of life and prevent further neurological deterioration in MC. To the best of our knowledge, gadobutrol-enhanced MRI has not been reported in the diagnosis of MC. In this study, we present two cases of MC detected by gadobutrol-enhanced MRI and give a choice that may facilitate early diagnosis.

## Case presentation

### Case 1

**Fig. 1:**
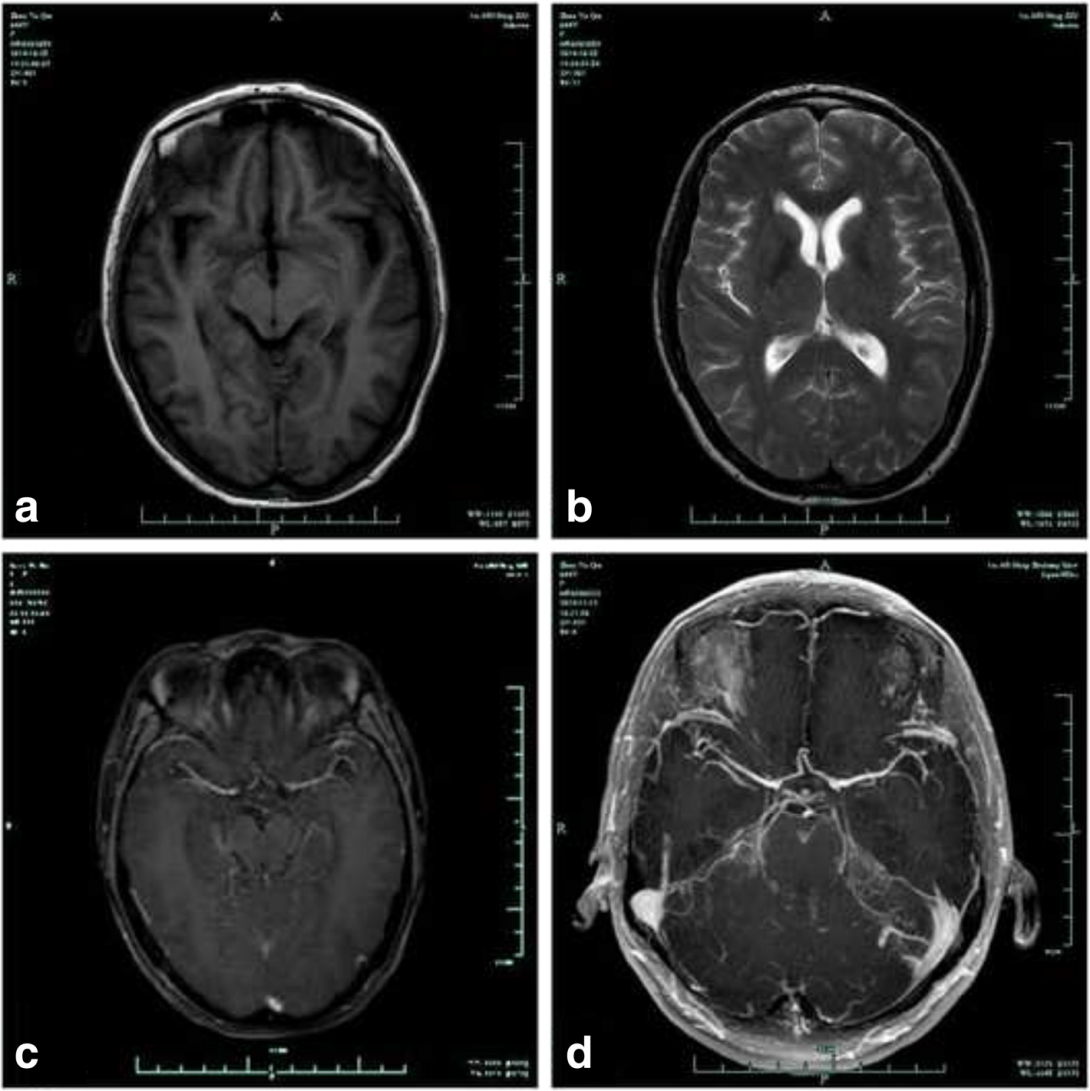
Axial magnetic resonance imaging of brain: T1-weighted imaging (**a**), T2-weighted imaging (**b**) and Gadopentetate dimeglumine-enhanced T1-weighted image (**c**) do not detect any solid tumor or enhancement meninges. While the gadobutrol-enhanced T1-weighted image shows serpentine enhancement along the surface of the brain (**d**)

A 44-year-old female was transferred to our department with a 1-month history of headache, nausea and vomiting. The patient had been diagnosed with cervical cancer and had undergone radical cervix resection followed by 6-period chemotherapy 1 year before admission. Gadopentetate dimeglumine-enhanced MRI of the brain (Philips Achieva 3.0 T Dual MRI, 0.1 mmol/kg, immediately after injection) was performed and did not detect any solid tumor (Fig 1a, b, c). Lumbar puncture was performed repeatedly, and headache could be temporary alleviated every time. Opening pressure increased from 200 to 350 mmH_2_O, but CSF cytology showed no evidence of malignant cells. Her CSF carbohydrate antigen (CA) 12-5, carbohydrate antigen 19-9 (CA19-9) were 976.7U/mL, 126.7U/mL compared to 1087.2U/mL and 130.9U/mL in serum. Chest and abdomen computed tomography (CT) scan showed no evidence of abnormality. Two weeks after admission, the patient had frequent tonic-clonic seizures, and complained of severe headache. Multidisciplinary teamwork recommended repeated CSF cytology examination, and brain MRI scan using gadobutrol (gadolinium-DO3A-butriol, Gadovist 1.0; Schering, Berlin, Germany) as contrast agent. Gadobutrol is not covered by patient’s medical insurance in our hospital and patients need to pay for it. As a result, we chose the gadopentetate dimeglumine which is in the range of medical insurance as the initial contrast agent for MRI scan. Finally the gadobutrol-enhanced MRI (Philips Achieva 3.0 T Dual MRI, 0.1 mmol/kg, immediately after injection) detected the meningeal metastasis (Fig 1d), and supported the diagnosis of meningeal carcinomatosis. Unfortunately the patient’ condition deteriorated very quickly and died 10 days later.

### Case 2

**Fig. 2:**
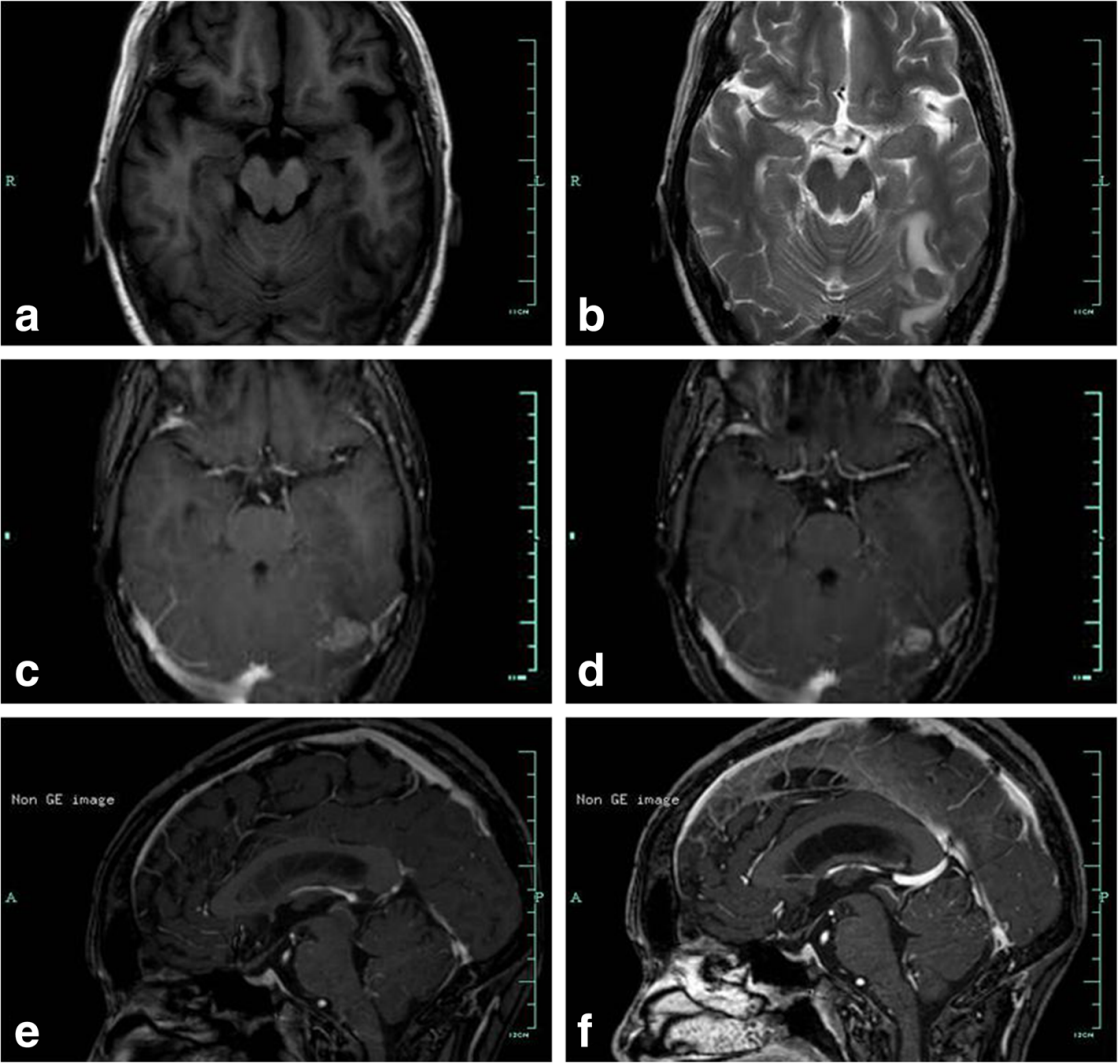
Gadopentetate dimeglumine-enhanced MRI scan shows an ovoid mass with the diameter of 1.6 centimeter in the left occipital lobe. The mass is hyperintense on T1-weighted image (**a**), iso- to hypointense on T2-weighted image (**b**), evident homogeneous enhancement on Gadopentetate dimeglumine-enhanced (**c**) and gadobutrol-enhanced (**d**) T1-weighted image. Gadobutrol-enhanced T1-weighted image detects nodular enhancement around the cerebellum (**f**) while gadopentetate dimeglumine-enhanced T1-weighted image shows no abnormality (**e**)

A 53-year-old female was referred to our department from a local hospital for further diagnosis and treatment after being diagnosed with a space-occupying lesion in the left occipital lobe. The patient presented with headache accompanied by nausea and vomiting for 10 days. In 2010, she had been diagnosed with and resected for gastric adenocarcinoma. Gadopentetate dimeglumine-enhanced MRI scan (Philips Achieva 3.0 T Dual MRI, 0.1 mmol/kg, immediately after injection) was performed to identify the occipital lesion. On the MRI scan, an ovoid mass with the diameter of 1.6 centimeter can be seen in the left occipital lobe. The mass was hyperintense on T1-weighted images, and iso- to hypointense on T2-weighted images (Fig 2a, b). After administration of meglumine gadopentetate, evident homogeneous enhancement was seen (Fig. 2c). The patient was diagnosed metastatic left occipital tumor from gastric adenocarcinoma, and Gamma knife surgery (GKS) with the marginal dose of 22 Gy was given. Three days after GKS, the patient had one time seizure of absences, and complained of aggravation of the headache. A brain CT scan showed no evidence of the tumor stroke. Considering the possibility of meningeal carcinomatosis, lumbar puncture was performed, but CSF cytology showed no evidence of malignant cells. Gadobutrol-enhanced MRI (Philips Achieva 3.0 T Dual MRI, 0.1 mmol/kg, immediately after injection) was performed, and meningeal disseminations around the cerebellum were detected (Fig 2f) except for the evident homogeneous enhancement mass in the left occipital lobe (Fig 2d). Therefore, a diagnosis of meningeal carcinomatosis was made. Radiotherapy and chemotherapy were recommended, while the patient chose medical treatment and died 2 months later.

## Conclusions

### A time course chart summarizing key events of the two cases is shown in the Fig. 3

**Fig. 3:**
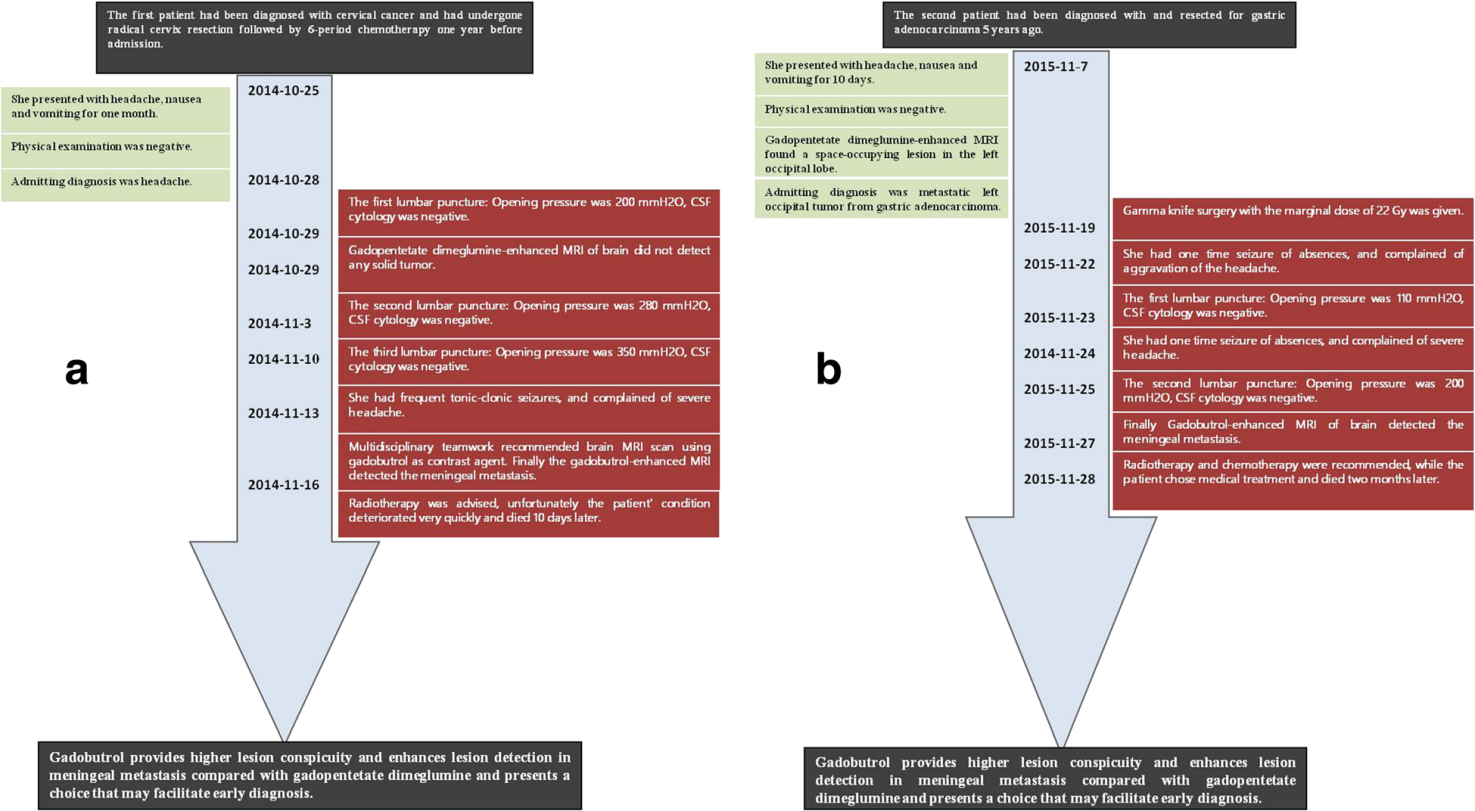
Time course chart of the first (**a**) and second case (**b**). Key events are summarized

Meningeal carcinomatosis is the last stage of patients with malignant tumors. Basically, CSF dissemination may happen in all patients with a malignant tumor but is most common in patients affected by lung cancer, breast carcinoma, melanoma or hematologic neoplasms such as lymphoma and leukemia [4]. The two patients both had malignant tumors, one with cervical cancer and another with gastric adenocarcinoma. Recent studies have suggested that the incidence of MC is higher than previously reported owing to the increasing of patients with advanced cancer and the development of neuroradiographic examination with gdMRI. However, timely diagnosis of MC may be challenging even for expert neurologists.

The clinical presentations associated with MC are symptoms mainly due to increased intracranial pressure such as headache, nausea and vomiting, neck pain as well as confusion. The two patients in this study all presented progressive headache from the early stage to the end and the opening pressure of the first patient increasing from 200 to 350 mmH_2_O was consistent with her headache. The headache progresses quickly and is usually insufferable in the last stage until to cerebral herniation [5]. In view of the feature, the diagnosis of MC could keep in mind and further examination such as CSF cytology and brain MRI should be arranged to identify the disease. Cranial nerve palsies resulting in diplopia, facial paralysis can also occur. These non-specific symptoms may be the initial signs in patients without tumor history [6]. Therefore the diagnosis of MC sometimes becomes extremely difficult in the early stage. Epilepsy appears to be one of the warning signals of deterioration. In our study, both patients had seizure in the last stage and deteriorated quickly until to death.

Brain gdMRI plays a growing role in the diagnosis of MC especially when CSF cytology is negative [7]. Postcontrast T1-weighted image (T1WI) is the most sensitive MRI sequence for detecting MC. The typical appearance of MC is serpentine, nodular, or plaque-like enhancement within the CSF and along the surfaces of the brain, spinal cord, and nerve roots. MRI findings are abnormal in 75–90 % patients with cytology-positive CSF [8]. Most experts agree that typical MRI findings in conjunction with a consistent clinical picture fulfill the diagnostic criteria for MC. However, most patients are diagnosed in the last stage especially when all the other examinations have been given. Therefore, several studies have focused on improving the efficiency of the brain MRI, and on introducing new potentially useful MRI subsequences [2, 9]. Gadobutrol is a new extracellular contrast agent which belongs to non-ionic macrocyclic gadolinium chelate. The lower osmolality and viscosity of gadobutrol enables the double-concentrated solution, which contains twice the amount of Gd chelate per volume. The T1 relaxivity of gadobutrol is approximately 14–27 % higher than that of other 0.5-mol/L Gd chelates [10], such as gadopentetate dimeglumine. Quantitative analysis revealed gadobutrol provided better image quality than conventional MR contrast agent based on the same dose and administered over the same time interval. In this study, we present two cases where MC was identified on gadobutrol-enhanced MRI but not enhancing on the traditionally gadopentetate dimeglumine-enhanced MRI. Kim et al. [10], also demonstrated that gadobutrol provided higher lesion conspicuity and enhances lesion detection in brain metastasis compared with gadopentetate dimeglumine. These findings may be a cue to analyze the accuracy in the diagnosis of MC, and present a choice that may facilitate early diagnosis. However, these findings need to be externally validated and its clinical value has to be verified with additional clinical studies.

The gold diagnosis standard is CSF cytology with the specificity of about 95 % for MC [6]. However, nearly 50 % patients are cytologically negative on the first lumber puncture examination. Repeat lumbar puncture can improve the result but little benefit is obtained after three punctures. In the study reported by Clarke et al. [11], 65 % of patients had CSF analysis and 28 % of them had positive finding. Considering the low positive rate, many studies have focused on some technique which can elevate the sensitivity of CSF examination. Subira et al. studied the diagnostic significance of flow cytometry immunophenotyping (FCI) in patients with MC, and found that FCI showed greater sensitivity and negative predictive value (79.79 % and 68.85 respectively) [8, 12]. Emilie et al. studied the validity of CSF CA15-3 using an automatized immune-enzymatic technology in breast cancer-related MC and a cut-off of 3 UI/ml for CSF CA15-3 with 80 % sensitivity and 70 % specificity was determined [13]. The first patient in our study also shows high level of CSF CA12-5 and CA19-9.

MC is usually fatal for patients with advanced cancer and the median survival ranges from 4 to 6 weeks. Considering the poor prognosis early intervention may prolong survival and relieve symptoms for patients. Radiotherapy and chemotherapy are the main therapeutic approaches, and the supportive care is also very important for patients. Because the studies of MC are small clinical series, retrospective analyses or clinical experience, there are no clear guidelines which treatments fits best for each patient.

Although rare, MC should be considered a possible diagnosis when the patient presents with progressive headache and further examination such as CSF cytology and brain MRI should be arranged to identify the disease. Brain gdMRI plays a growing role in the diagnosis of MC especially when CSF cytology is negative. Gadobutrol provides higher lesion conspicuity and enhances lesion detection in meningeal metastasis compared with gadopentetate dimeglumine. These findings may be a cue to analyze the accuracy in the diagnosis of MC, and present a choice that may facilitate early diagnosis.

## Abbreviations

CA: Carbohydrate antigen
CSF: Cerebrospinal fluid
CT: Computed tomography
FCI: Flow cytometry immunophenotyping
gdMRI: Gadolinium magnetic resonance imaging
GKS: Gamma knife surgery
MC: Meningeal carcinomatosis
T1WI: T1-weighted image
